# Supplementary material for: Temporal analysis of genetic diversity and gene flow in the threatened catfish Pseudoplatystoma magdaleniatum from a dammed neotropical river
Source: PLoS One. 2024 Apr 18;19(4):e0301577. doi: 10.1371/journal.pone.0301577 (PMC11025948; doi:10.1371/journal.pone.0301577)
Supplement: Attachment — Submitted filename: Garcia-Castro and Marquez 1.pdf [file pone.0301577.s002.pdf]

Abstract

The striped catfish *Pseudoplatystoma magdaleniatum* is a large-size migratory species from north Andes, endemic to Magdalena basin and the main fishing resource behind bocachico *Prochilodus magdalenae*. Despite the estimated reduction of over 80 % of the fisheries production of this species throughout the basin in recent decades, its population in the lower Magdalena-Cauca basin showed healthy genetics. However, the current conservation status of this species and several habitat disturbances demand the evaluation of its population genetics to infer evolutionary risks and measure potential changes. This work analyzed a total of 164 samples from the Cauca River collected downstream of the Ituango dam between 2019 – 2021, using species-specific microsatellite markers to compare its genetic diversity and structure in samples collected between 2010 – 2014 from the lower Magdalena-Cauca basin. Our results showed a relatively stable panmictic population over time (4 to 10 years), with high genetic diversity and evidence of recent bottleneck. Some actions such as prioritizing the habitat connectivity of the striped catfish to conserve its gene flow, the exploration of a greater geographic range to characterize its diversity and genetic structure in the rest of the basin, and the integration of these results with future monitoring, are important measures to consider in management plans for *P. magdaleniatum* in the Magdalena-Cauca basin.

Introduction

The Cauca River is the main tributary of the Magdalena Basin at north Andes, subjected to strong anthropic pressures because of the settling of a large part of the Colombian population and the support of the local economy (Restrepo *et al.*, 2020). The greatest threats to the Magdalena-Cauca basin are mainly related to mining, water pollution and habitat modification by agriculture, livestocking, deforestation and dam constructions (López-Casas *et al.*, 2020; Restrepo *et al.*, 2020). These and other factors, such as overfishing, introduction of exotic species and genetic contamination of populations (Márquez *et al.*, 2020), are of growing concern due to their potential impacts on biodiversity. In particular, the Magdalena River basin has 233 fish species (14.5% of the country's freshwater ichthyofauna), 68.1% of which are endemic and more than 15% are included in the red list of freshwater fishes of Colombia with some degree of threat (García-Alzate *et al.*, 2020; Mojica *et al.*, 2012).

One of these endemic species is the striped catfish *Pseudoplatystoma magdaleniatum*, a large migratory fish and the most important fishing resource of the country after the bocachico *Prochilodus magdalenae*, with 11% of the total landings in the Magdalena basin in 2019 (Valderrama *et al.*, 2020). However, it is estimated that its fishery production has been reduced by around 90 % since 1970, which is the main cause of *P. magdaleniatum* being classified Endangered or Critically Endangered

in national and international red lists (Mojica *et al.*, 2016; Mojica *et al.*, 2012). The population of this species in the middle and lower sectors of the Magdalena-Cauca basin showed the same gene pool and high genetic diversity (García-Castro *et al.*, 2021). Contrastingly, other migratory species of high commercial interest such as *Pimelodus yuma* (nicuro), *Pimelodus grosskopfii* (barbudo) and *Prochilodous magdalenae* (bocachico) with gene flow in the middle and lower sectors of the Cauca River have showed high degree of inbreeding (see Márquez *et al.*, 2020).

Since the fragmentation of rivers by dam constructions generates great uncertainty due to the natural landscape modification and its potential impacts on migratory rheophilic species (Barbarossa *et al.*, 2020; Dudgeon *et al.*, 2006; Liermann *et al.* al., 2012; Reid *et al.*, 2019), the hydroelectric project Hidroituango in the Cauca River arises among multiple factors that potentially threaten the biodiversity of this basin (Jiménez-Segura *et al.*, 2014, 2016; López -Casas *et al.*, 2020; Márquez *et al.*, 2020). This dam (here called Ituango dam) is in the Cauca River canyon, an area of rapids and slopes that naturally limit the upstream migration of species such as *P. magdaleniatum*, so the populations of this fish are mainly found downstream site of this hydroelectric (García-Alzate *et al.*, 2020; Rodríguez-Olarte *et al.*, 2011). However, although the migratory route of *P. magdaleniatum* is not considered interrupted, potential downstream effects that may disturb the migration behavior of this species might have impacts at population level, as migration is a key factor in the evolutionary processes of wild populations (Gagnaire *et al.*, 2015; Hedgecock *et al.*, 2007; Martinez *et al.* 2018). For instance, sediment retention upstream can alter the balance in sedimentation levels and reduce the availability of nutrients downstream (Kemp *et al.*, 2011, Kondolf *et al.*, 2014), especially in floodplains, which are crucial places for the life cycle of migratory fishes in the basin (Jiménez-Segura *et al.*, 2016). Similarly, the modification of the water flow due to the regulation of discharges can alter the endocrine response associated with the reproductive migration of fish downstream, as it has been reported in *P. magdalenae* from La Miel River (Fex-Wolf *et al.*, 2019). Other factors such as the increasing of organic mercury levels and the pH and oxygen alteration downstream, because of trophic and biochemical effects that occur mainly upstream, are more particular impacts that depend on the characteristics of the dam and the river system (Agostinho *et al.*, 2008; Forsberg *et al.*, 2017).

However, knowledge about the real impacts of the above factors on population genetics of non-fragmented species is quite limited, whereas the few studies that report some degree of genetic change on a temporal scale in populations located downstream of the dams constitute cases where migratory routes are effectively fragmented and those changes are generally associated with a reduced effective population size or bottleneck events that might be related to that isolation (*e. g.* Hansen *et al.*, 2014; Liu *et al.*, 2020; Vega-Retter *et al.*, 2020; Yamamoto *et al.*, 2004). Furthermore, considering the

multiple threats to biodiversity in the Magdalena-Cauca basin, genetic monitoring emerges as an alternative to estimate the status and potential changes of wild populations over time, mainly of those species with some degree of conservation concern and subjected to changes in its ecosystem (Laikre *et al.*, 2010; Schwartz *et al.*, 2007). Therefore, this study analyzed the population genetics of *P. magdaleniatum* on a time scale, using samples collected in the Magdalena-Cauca basin before (Ex-ante; 2010 - 2014) and after (Ex-post; 2019 - 2021) the construction of the Ituango dam, using species-specific microsatellite markers.

The expectations of this work were to find a population with high genetic diversity and gene flow in the Cauca River, based on the findings reported by García-Castro *et al.* (2021) of the Ex-ante sample. Additionally, despite the genetic evidence of a reduced population size (García-Castro *et al.*, 2021) and the persistence of different anthropic pressures on this species (López-Casas *et al.*, 2020), it is not expected to obtain substantial genetic changes in terms of diversity or structure in the population of *P. magdaleniatum* on the considered time scale. These two hypotheses are mainly based on: (1) the absence of fragmentation of the population of *P. magdaleniatum* in the Cauca River, (2) the short period of time separating the Ex-ante and Ex-post samples in relation to the generation length of this species (approximately four years; Mojica *et al.*, 2016), and (3) the delay that may exist between contemporary demographic/environmental processes and the expression of their impact on the genetic diversity and structure (Epps & Keyghobadi, 2015; Landguth *et al.*, 2010).

Materials and Methods

Sampling and genotyping

The Cauca River runs along 1,350 km from south to north in the Colombian Andes, from the Alto Cauca valley between 3,200 and 1,000 m.a.s.l. to the Cauca canyon in the middle and lower sectors of the basin, a geography with natural barriers for many migratory species such as *P. magdaleniatum* and where the current Ituango dam of the Hidroituango hydroelectric project is located. In the last 500 km, the Cauca River receives important tributaries such as the Ituango and Nechí rivers and feeds several floodplains during raining periods until it joins the Magdalena River in the Mompós Depression. This last section has an important part of the fish biodiversity of the Magdalena-Cauca basin and concentrates the most commercially important fishing resources (Galvis & Mojica, 2007; García-Alzate *et al.*, 2020).

It was analyzed a total of 164 muscle or fin tissues from individuals of *P. magdaleniatum* collected between the years 2019 – 2021, called Ex-post sample from now on, in eight sectors of the middle and lower part of the Cauca River (S2, S3, S4, S5, S6, S7 and S8; see Landínez-García & Márquez,

2016), located downstream of the Ituango dam. These samples were supplied by Grupo de Ictiología from Universidad de Antioquia (GIUA), Fundación Humedales and Grupo de Biotecnología Animal from Universidad Nacional de Colombia, Sede Medellín. Due to low and heterogeneous sample numbers among sectors, the samples were grouped into three larger sectors denoted as: S2-S3 (N= 49), S4-S5 (N= 59) and S6-S7-S8 (N= 56), for population genetic analyzes (Fig 1).

Fig 1. Sampling sites (circles) of *Pseudoplatystoma magdaleniatum* in three sectors of the Cauca River downstream the Ituango dam.
S2-S3: Ituango River mouth, Golondrina, Espíritu Santo River mouth, Puerto Valdivia; S4-S5: Puerto Jardín, Man River, El Doce, Cáceres, Caucasia, La Ilusión, Nechí, Palomar; S6-S7-S8: Guaranda, Tres Cruces and floodplains La Raya, La Panela, Piqué and El Floral.

The DNA extraction was performed using the commercial GeneJet Genomic DNA Purification Kit (Thermo Scientific). The microsatellite regions were amplified using 13 primers previously designed and evaluated in wild populations of *P. magdaleniatum* (Psm03, Psm04, Psm06, Psm11, Psm14, Psm16, Psm18, Psm19, Psm21, Psm22, Psm24, Psm25 and Psm26) and the same conditions of PCR reported by García-Castro *et al.* (2021). The fragments were separated by capillary electrophoresis on the ABI 3730 XL automatic sequencer (Applied Biosystems), using LIZ600 (Applied Biosystems) as an internal molecular size marker. Alleles were recorded in GeneMarker software v.3.0.0 and amplification and scoring errors were evaluated in Micro-Checker v.2.2.3 (Van Oosterhout *et al.*, 2004).

Genetic diversity and demographic events

To estimate the genetic diversity of the Ex-post sample, the average number of alleles per locus (Na), allelic range (Ra) and the expected (H_E) and observed (H_O) heterozygosities were calculated using the GenAlEx v6.51b2 program (Peakall & Smouse, 2006, 2012) and the allelic richness (Ar) was calculated in FSTAT v.2.9.4 (Goudet, 2003). Inbreeding coefficients (F_{IS}) and deviations from Hardy-Weinberg (HWE) and linkage (LD) equilibria were evaluated in Arlequin v3.5.2.2 (Excoffier & Lischer, 2010). The multilocus significance values for the HWE by population or sector were calculated using the Fisher's Exact Test integrated in the web version of GENEPOP v4.7.5 (Raymond & Rousset, 1995; Rousset, 2008).

Drastic reduction in population size (bottleneck) was evaluated using the excess heterozygosity test with the BOTTLENECK software v1.2.02 (Piry *et al.*, 1999) and its default parameters, comparing three likely mutation models in microsatellites (Cornuet & Luikart, 1996): infinite alleles model

(IAM), stepwise mutation model (SMM) and two-phase mutation model (TPM). A second approach consisted of calculating the standardized M index of Garza and Williamson (2001) in Arlequin v3.5.2.2 (Excoffier & Lischer, 2010), which determines that the reduction in the number of alleles with respect to the allelic range of a population can detect recent bottleneck events. Additionally, the effective population size (Ne) was estimated in NeEstimator v2.1 (Do *et al.*, 2014) using the linkage disequilibrium (LD) method and evaluating allele frequencies greater than or equal to 0.02, since lower allelic frequencies tend to overestimate the Ne (Do *et al.*, 2014; Waples & Do, 2010). Finally, an analysis to detect the most likely first-generation migrants (individuals from another sampled sector) was used in the GENECLASS2 software (Piry *et al.*, 2004), implementing a Bayesian method (Baudouin & Lebrun, 2001) and the unbiased Monte Carlo resampling method (Paetkau *et al.*, 2004), with 13 loci, 10,000 individuals and a significance level of 0.01. In addition, for analyzes with multiple comparisons, the Bonferroni correction was applied.

Genetic structure

For analysis of the genetic structure in the Ex-post sample, pairwise comparisons of the standardized indices F'_{ST} (Meirmans, 2006; Meirmans & Hedrick, 2011) and Jost's D_{EST} (Jost *et al.*, 2018; Meirmans & Hedrick, 2011) were used and an analysis of molecular variance (AMOVA; (Meirmans, 2006) was performed, using GenAlEx v6.51b2 (Peakall & Smouse, 2006, 2012). Genetic differentiation between sectors was also evaluated through a discriminant analysis of principal components (DAPC) using the R package Adegenet (Jombart, 2008). Finally, a Bayesian clustering analysis was performed on Structure v2.3.4 (Pritchard *et al.*, 2000) using 800,000 Markov chain Monte Carlo (MCMC), 80,000 of these as burn in, and the models of LOCPRIOR, genetic mixture and correlated alleles. It was evaluated at K = 1 to K = m + 3, where m is the number of *a priori* populations (Evanno *et al.*, 2005), with 20 repeats each. Then, to determine the most likely number of populations (K), the StructureSelector software (Li & Liu, 2018) was used to calculate six statistics (MedMeaK, MaxMeaK, MedMedK and MaxMedK: Puechmaille, 2016; Ln Pr (X | K): Pritchard *et al.*, 2000; and ΔK: Evanno *et al.*, 2005) and plot the histogram of co-ancestry probabilities of all individuals.

Comparative analysis of temporal samples

To explore genetic changes on a temporal scale of the population of this species, 147 genotypes (Ex-ante sample) were included in the analysis (years 2010 - 2014, sectors of the middle and lower sites of the Magdalena-Cauca basin; see García-Castro *et al.*, 2021), for a total of 311 individuals of *P. magdaleniatum*. The genetic diversity (Na, Ar, Ra, H_E, H_O, F_{IS}, HWE), demographic events (evaluation of bottlenecks and estimation of Ne) and genetic structure between the Ex-ante and Ex-post samples of *P. magdaleniatum* were evaluated using the above methodology and descriptive

comparisons. Additionally, a genic differentiation test (G test) was carried out in the web version of GENEPOP v4.7.5 (Raymond & Rousset, 1995; Rousset, 2008), which evaluate the pairwise differences of the allele's distribution of each locus between the temporal samples, using the modified Fisher Exact Test to evaluate statistical significance.

Finally, the effective population size was estimated using the temporal method (two or more temporally separated samples) in NeEstimator v2.1 (Do *et al.*, 2014) as an alternative to the LD method (single sample). For this, a minimum separation of two generations between the samples was assumed based on a generation length of about four years for *P. magdaleniatum* (Mojica *et al.*, 2016). Only the Fs value was considered as an estimator of the change in allelic frequencies, which is less biased than its analogues Fc and Fk, although less precise (Jorde & Ryman, 2007). The Plan II was considered as the sampling method, which does not require knowing the population size (N) to use it as a parameter for the estimation of Ne and assumes that individuals are collected before reproducing and without returning to the original population (Do *et al.*, 2014).

Results

Genetic diversity

Genotyping errors as null alleles were not detected, and stuttering effects were corrected when present. Genetic diversity in the three sectors was high, with similar values of average numbers of alleles per locus (Na), allelic richness (Ar), allelic range (Ra) and expected (H_E) and observed (H_O) heterozygosities (Table 1). Inbreeding coefficients (F_{IS}) were positive (0.003 - 0.021), but not significant ($P > 0.05$).

Table 1. Average values per locus of genetic diversity of *Pseudoplatystoma magdaleniatum* in the Ex-post (collected between years 2019 – 2021 in three sectors of the Cauca River downstream of the Ituango dam) and Ex-ante (years 2010 – 2014; García-Castro *et al.*, 2021) samples.

Sample	N	Na	Ar	Ra	H_O	H_E	P	F_{IS}
S2-S3	49	9.154	8.820	36.308	0.753	0.771	0.249	0.004
S4-S5	59	10.154	9.461	41.231	0.776	0.785	**0.042**	0.003
S6-S7-S8	56	8.846	8.497	34.769	0.756	0.771	**0.023**	0.021
Overall (Ex-post)	164	11.000	10.762	43.077	0.762	0.777	**0.007**	0.023
Ex-ante	147	11.308	11.162	44.615	0.765	0.783	0.094	-0.019

N: sample size, Na: number of alleles, Ar: allelic richness, Ra: allelic range, H_E: expected heterozygosity and H_O: observed heterozygosity, F_{IS}: inbreeding coefficient, P: p-value of the Hardy Weinberg equilibrium test. Values in bold denote statistical significance.

On the other hand, sectors S4-S5 and S6-S7-S8 and the overall Ex-post sample presented Hardy-Weinberg disequilibrium (P: 0.042, 0.023 and 0.007, respectively). However, no locus presented deviation from equilibrium in S4-S5 and only one (Psm18) presented deviation in S6-S7-S8, so this result can be attributed to false positives of the applied multilocus significance test (Fisher's Exact test).

At temporal scale, the Ex-post sample showed slightly reduced genetic diversity (Na, Ar, Ra, H_O and H_E) respect to the Ex-ante sample, as well as increased inbreeding coefficient, although this last was non-significant in both temporal samples (Table 1). Indeed, only one of the loci (Psm24) showed differences in its allelic distributions between the two samples (P = 0.012), so that in general, the allelic distributions did not evidence variations over time (P multilocus = 0.077).

Demographic events

The excess heterozygosity test to detect recent bottleneck events was significant (P <0.017) in the three sectors and overall (Ex-post) using the IAM, which is considered one of the most likely mutation models in microsatellites (Cornuet & Luikart, 1996; Piry *et al.*, 1999). Additionally, the M indices were lower than the reference value of 0.68 (Garza & Williamson, 2001). These results together indicate a recent reduction in the population size of *P. magdaleniatum* in the Cauca River, same as the Ex-ante sample (Table 2).

Table 2. Estimation of recent bottleneck events and effective population size in Ex-post (2019 – 2021, sectors S1 - S8 of the Cauca River) and Ex-ante (years 2010 – 2014; García-Castro *et al.*, 2021) samples of *Pseudoplatystoma magdaleniatum*.

Sample	M	IAM	TPM	SMM	Ne	CI
S2-S3	0.21	**0.001**	0.207	0.863	814.9	223.7 - ∞
S4-S5	0.23	**0.000**	0.108	0.936	∞	1008.2 - ∞
S6-S7-S8	0.21	**0.001**	0.122	0.554	∞	695.7 - ∞
Overall (Ex-post)	0.23	**0.000**	0.095	0.996	2,126.3	626.9 - ∞
Ex-ante	0.23	**0.000**	0.137	0.998	1379.4	414.4 - ∞

M: standardized index of Garza-Williamson (2001). IAM, TPM, SMM: p-values (in bold for statistical significance after Bonferroni correction) of the heterozygosity excess test implemented in BOTTLENECK v1.2.02 (Piry *et al.*, 1999) according to the assumed mutational model. Ne: effective population size (Ne) estimated using the Linkage disequilibrium (LD) method. CI: confidence interval of the Ne.

On the other hand, the effective population size using the LD method varied between 814.9 and ∞ in the three sectors. Estimates equal to ∞ can be fully explained by a sampling error that is greater than the genetic drift signal, so they do not provide evidence that the population is not very large (Waples & Do, 2010). This method showed that the overall estimation (Ex-post) was higher than that of the Ex-ante sample. These two values were close to that obtained using the temporal method: 1,745.3 (CI: 446.2 – ∞). Therefore, both methods suggest that the Ne of *P. magdaleniatum* is greater than 1,300 in both temporal samples, with the lowest confidence limit of 414 (Table 2).
Finally, the exploration of migratory events among sectors of the Ex-post sample using the GENECLASS2 software (Piry *et al.*, 2004), detected three individuals collected in S4-S5 as likely immigrants (P: 0.006, 0.005 and 0.009) from the lowest sector (S6-S7-S8), whereas another individual collected in the highest sector (S2-S3) was assigned to the lowest sector S6-S7-S8 (P = 0.006); supporting the gene flow throughout the studied area.

Genetic structure

Although the overall structure index calculated using the AMOVA was significant ($F_{ST\,(2,\,327)} = 0.014$; P = 0.013), geographical genetic structure for the Ex-post sample was fully explained by the variances among individuals within (77%) and at regional scale (23%). The absence of geographical genetic structure was supported by both F'_{ST} and Jost´s D_{EST} indices (Table 3) and by the discriminant analysis of principal components (DAPC; Fig 2A), which showed an overlapping of the three sectors. Additionally, although the estimators of the best K from the Bayesian analysis differed, suggesting K = 1 (Ln Pr (X | K)), K = 2 (MedMedK, MedMeanK, MaxMedK, MaxMeanK) and K = 5 (ΔK), the homogeneous distribution of the co-ancestry probabilities along the sampling area when 2 ≤ K ≤ 3 (Fig 2B-2C) supports both the absence of geographical genetic structure and the presence of a single genetic stock.

Table 3. Pairwise comparison of the standardized genetic structure indices F'_{ST} (below the diagonal) and Jost's D_{EST} (above the diagonal) in the Ex-post sample (collected between 2019-2021, sectors S1 - S8 in Cauca River).

Sitio	S2-S3	S4-S5	S6-S7-S8
S2-S3	-	0.001	0.008
S4-S5	0.012	-	0.007
S6-S7-S8	0.017	0.012	-

No statistical significance was found after correction for multiple comparisons.

Fig 2. (A) Discriminant analysis of principal components (DAPC; 30 principal components retained and 71.3% of the variance) and co-ancestry probabilities when (B) K = 2 and (C) K = 3 of 164 individuals of *Pseudoplatystoma magdaleniatum* of the Ex-post sample (2019-2021, sectors S2 - S8 of the Cauca River).

Finally, no differences were detected in the genetic structure when comparing the Ex-ante and Ex-post samples based on Jost's D_{EST} (D_{EST} = 0.001, P = 0.351), the AMOVA (0% of the variance between samples), the DAPC (Fig 3A) and the Bayesian analysis (Fig 3B; K = 1 according to the estimators Ln Pr (X | K), MedMedK, MedMeanK, MaxMedK and MaxMeanK), only differing in the F'_{ST} index (F'_{ST} = 0.009, P = 0.007).

Fig 3. (A) Discriminant analysis of principal components (DAPC; 50 principal components retained and 85% of the variance) and (B) co-ancestry probabilities of 311 individuals of *Pseudoplatystoma magdaleniatum* (147 Ex-ante: years 2010-2014, middle sectors and lower of the Magdalena-Cauca basin; 164 Ex-post: years 2019-2021, sectors S1 - S8 of the Cauca River).

Discussion

Factors supporting the need for a continue genetic evaluation of wild populations are related to environmental and anthropogenic pressures that generate changes in the landscape and threaten the balance of the species, particularly those with some degree of vulnerability (Laikre *et al.*, 2010; Schwartz *et al.*, 2007). In this work, 13 species-specific polymorphic loci previously developed and evaluated in the population genetics of *P. magdaleniatum* were used for genetic evaluation of 164 individuals (Ex-post sample, years 2019 – 2021) distributed in three sectors of the Cauca River downstream of the Ituango dam.

The genetic diversity of the Ex-post sample was high, with H_E values higher than both the average of Neotropical catfishes (H_E: 0.609 ± 0.210; see Hilsdorf & Hallerman, 2017) and values reported in some populations of other species of the genus *Pseudoplatystoma* (Abreu *et al.*, 2009; Dantas *et al.*,
