# Supplementary material for: Temporal analysis of genetic diversity and gene flow in the threatened catfish Pseudoplatystoma magdaleniatum from a dammed neotropical river
Source: PLoS One. 2024 Apr 18;19(4):e0301577. doi: 10.1371/journal.pone.0301577 (PMC11025948; doi:10.1371/journal.pone.0301577)
Supplement: Attachment — Submitted filename: Garcia-Castro and Marquez 2.pdf [file pone.0301577.s003.pdf]

2013; Pereira *et al.*, 2009; Prado *et al.*, 2018). Additionally, this genetic diversity was like that of the Ex-ante sample (years 2010 – 2014) from the middle and lower sectors of the Magdalena-Cauca basin (García-Castro *et al.*, 2021), supporting the initial hypothesis of this study about the stability of its genetic diversity over time. Since the comparisons of genetic diversity are mainly descriptive and no differences were detected in the allelic frequency distribution between the temporal samples (G test, $P > 0.05$), the observed differences may result from stochastic effects inherent to sampling (Epps & Keyghobadi, 2015; Landguth *et al.*, 2010).

An important indicator that measures the evolutionary potential of populations is the effective size (Ne), which can be estimated using a single sample in time (e.g., linkage disequilibrium method) or several samples separated by n generations (temporal method). Both approaches were performed in this work, finding an increase in Ne in the Ex-post sample using the LD method. However, some factors such as the presence of individuals of different generations (age structure) or high levels of immigration in the population can bias such estimation (Waples *et al.*, 2014; Waples & England, 2011), which could explain differences between Ex-ante and Ex-post samples. However, these estimations were like that obtained using the temporal method, which generates a single value applicable to all the generations assumed between the samples. This is consistent with previous results of simulated data that demonstrate a similar performance of these two methods when it is assumed few generations (Waples & Do, 2010). The most recent recommendations for the conservation of the evolutionary potential of species in wild populations suggest values of $Ne \geq 1,000$ (Frankham *et al.*, 2014). Although the estimates obtained in this study are higher than 1,300, the confidence intervals show the minimum values of 414 that implies evolutionary risks for the species (Frankham *et al.*, 2014), so we suggest considering this last value in the first instance to support management measures that prevent the genetic erosion of *P. magdaleniatum*, considering its current conservation status.

On the other hand, our results show the absence of geographical genetic structure in the Ex-post sample and the presence of the same single genetic stock from the middle and lower sectors of the Magdalena-Cauca basin. This supports the initial hypothesis about the prevalence of a panmictic population (random reproduction) with gene flow along the Cauca River in *P. magdaleniatum*. The absence of spatial barriers downstream of the Ituango dam in the Cauca River and extensive floodplains that remain in the lower part of the Magdalena-Cauca basin would allow large dispersion of this species and connectivity of its population (Restrepo *et al.*, 2020; see López-Casas *et al.*, 2016). Although samples from the Magdalena River were not used in the Ex-post sample, it is likely that this species conserves gene flow between these rivers (see García-Castro *et al.*, 2021). This hypothesis should be tested in future studies that include samples of the Magdalena River, particularly of its

upper sites and the rest of its tributaries such the San Jorge River, which would allow to know whether this same genetic population is distributed in the rest of the basin.

In wild populations, the number of generations required to detect genetic response to perturbations (time lag) could depend on several factors such as the mutation rate, dispersal rate, population dynamics, the generation length of the species and the effective population size (see Epps & Keyghobadi, 2015). Therefore, a likely large dispersal rate, a large enough effective population size and a short time frame evaluated in this work, translated into a small number of generations between the temporal samples, could explain the stability or the undetected changes in both genetic diversity and structure found in *P. magdaleniatum* that support the proposed initial hypotheses.

In conclusion, our results indicate that *P. magdaleniatum* has the same genetic population along the Cauca River with high genetic diversity, although preserving evidence of recent reductions in its population size. Given that reduced populations are more prone to the effects of genetic drift and therefore to changes in genetic diversity (Garza & Williamson, 2001; Waples, 2015; Waples & Do, 2010), monitoring of populations with evidence of bottleneck is relevant. On the other hand, the conservation of dispersion and migratory behavior of this fish is crucial to maintain the gene flow found in its population, so ensuring the connectivity of the striped catfish´s habitat should be a priority in management plans in the long-term.

Acknowledgements

This study was supported by a grant framed under the Project "Variabilidad genética de un banco de peces de los sectores medio y bajo del río Cauca" (CT-2019-000661, Empresas Públicas de Medellín and Universidad Nacional de Colombia, Sede Medellín).

References

Abreu, M. M. de, Pereira, L. H. G., Vila, V. B., Foresti, F., & Oliveira, C. (2009). Genetic variability of two populations of *Pseudoplatystoma reticulatum* from the Upper Paraguay River Basin. *Genetics and Molecular Biology*, *32*(4), 868–873. https://doi.org/10.1590/S1415-47572009005000075

Agostinho, A. A., Pelicice, F. M., & Gomes, L. C. (2008). Dams and the fish fauna of the Neotropical region: Impacts and management related to diversity and fisheries. *Brazilian Journal of Biology*, *68*(4, Suppl.), 1119–1132. https://doi.org/10.1590/S1519-69842008000500019

Barbarossa, V., Schmitt, R. J. P., Huijbregts, M. A. J., Zarfl, C., King, H., & Schipper, A. M. (2020). Impacts of current and future large dams on the geographic range connectivity of freshwater

fish worldwide. *Proceedings of the National Academy of Sciences of the United States of America*, *117*(7), 3648–3655. https://doi.org/10.1073/pnas.1912776117

Baudouin, L., & Lebrun, P. (2001). An operational bayesian approach for the identification of sexually reproduced cross-fertilized populations using molecular markers. *Acta Horticulturae*, *546*(1), 81–93. https://doi.org/10.17660/actahortic.2001.546.5

Cornuet, J. M., & Luikart, G. (1996). Description and power analysis of two tests for detecting recent population bottlenecks from allele frequency data. *Genetics*, *144*(1), 2001–2014. https://doi.org/10.1093/oxfordjournals.jhered.a111627

Dantas, H. L., dos Santos Neto, M. A., Oliveira, K. K. C., Severi, W., Diniz, F. M., & Coimbra, M. R. M. (2013). Genetic diversity of captive and wild threatened catfish *Pseudoplatystoma corruscans* in the São Francisco River. *Reviews in Fisheries Science*, *21*(3–4), 237–246. https://doi.org/10.1080/10641262.2013.800787

Do, C., Waples, R. S., Peel, D., Macbeth, G. M., Tillett, B. J., & Ovenden, J. R. (2014). NeEstimator v2: Re-implementation of software for the estimation of contemporary effective population size (Ne) from genetic data. *Molecular Ecology Resources*, *14*(1), 209–214. https://doi.org/10.1111/1755-0998.12157

Dudgeon, D., Arthington, A. H., Gessner, M. O., Kawabata, Z. I., Knowler, D. J., Lévêque, C., Naiman, R. J., Prieur-Richard, A. H., Soto, D., Stiassny, M. L. J., & Sullivan, C. A. (2006). Freshwater biodiversity: Importance, threats, status and conservation challenges. *Biological Reviews of the Cambridge Philosophical Society*, *81*(2), 163–182. https://doi.org/10.1017/S1464793105006950

Epps, C. W., & Keyghobadi, N. (2015). Landscape genetics in a changing world: Disentangling historical and contemporary influences and inferring change. *Molecular Ecology*, *24*(24), 6021–6040. https://doi.org/10.1111/mec.13454

Evanno, G., Regnaut, S., & Goudet, J. (2005). Detecting the number of clusters of individuals using the software STRUCTURE: A simulation study. *Molecular Ecology*, *14*(8), 2611–2620. https://doi.org/10.1111/j.1365-294X.2005.02553.x

Excoffier, L., & Lischer, H. E. L. (2010). Arlequin suite ver 3.5: A new series of programs to perform population genetics analyses under Linux and Windows. *Molecular Ecology Resources*, *10*(3), 564–567. https://doi.org/10.1111/j.1755-0998.2010.02847.x

Fex-Wolf, D. de, López-Casas, S., & Jiménez-Segura, L. F. (2019). Hydropower effects on *Prochilodus magdalenae* (Prochilodontidae) reproduction: Evidence from endocrine response. *Revista MVZ Cordoba*, *24*(2), 7180–7187. https://doi.org/10.21897/rmvz.1606

Frankham, R., Bradshaw, C. J. A., & Brook, B. W. (2014). Genetics in conservation management: Revised recommendations for the 50/500 rules, Red List criteria and population viability analyses. In *Biological Conservation* (Vol. 170, pp. 56–63). Elsevier. https://doi.org/10.1016/j.biocon.2013.12.036

Gagnaire, P. A., Broquet, T., Aurelle, D., Viard, F., Souissi, A., Bonhomme, F., Arnaud-Haond, S., & Bierne, N. (2015). Using neutral, selected, and hitchhiker loci to assess connectivity of marine populations in the genomic era. *Evolutionary Applications*, *8*(8), 769–786. https://doi.org/10.1111/eva.12288

Galvis, G., & Mojica, J. I. (2007). The Magdalena River freshwater fishes and fisheries. *Aquatic Ecosystem Health & Management*, *10*(2), 127–139. https://doi.org/10.1080/14634980701357640

García-Alzate, C., DoNascimiento, C., Villa-Navarro, F. A., García-Melo, J. E., & Herrera R., G. (2020). Diversidad de peces de la cuenca de Río Magdalena, Colombia. In L. Jiménez-Segura & C. A. Lasso (Eds.), *XIX. Peces de la cuenca del río Magdalena, Colombia: diversidad, conservación y uso sostenible* (pp. 85–113). Serie Editorial Recursos Hidrobiológicos y Pesqueros Continentales de Colombia. Instituto de Investigación de Recursos Biológicos Alexander von Humboldt. https://doi.org/DOI: 10.21068/B2020RRHHXIX02

García-Castro, K. L., Rangel-Medrano, J. D., Landínez-García, R. M., & Márquez, E. J. (2021). Population genetics of the endangered catfish *Pseudoplatystoma magdaleniatum* [illegible]

(Siluriformes: Pimelodidae) based on species-specific microsatellite loci. *Neotropical Ichthyology*, *19*(1). https://doi.org/10.1590/1982-0224-2020-0120

Garza, J. C., & Williamson, E. G. (2001). Detection of reduction in population size using data from microsatellite loci. *Molecular Ecology*, *10*(2), 305–318. https://doi.org/10.1046/j.1365-294x.2001.01190.x

Goudet, J. (2003). *Fstat (ver. 2.9.4), a program to estimate and test population genetics parameters*. https://www2.unil.ch/popgen/softwares/fstat.htm

Hansen, M. M., Limborg, M. T., Ferchaud, A. L., & Pujolar, J. M. (2014). The effects of medieval dams on genetic divergence and demographic history in brown trout populations. *BMC Evolutionary Biology*, *14*(1), 1–14. https://doi.org/10.1186/1471-2148-14-122

Hedgecock, D., Barber, P. H., & Edmands, S. (2007). Genetic approaches to measuring connectivity. *Oceanography*, *20*(3), 70–79. https://doi.org/10.5670/oceanog.2007.30

Hilsdorf, A. W. S., & Hallerman, E. M. (2017). Characterization of Genetic Resources. In A. W. S. Hilsdorf & E. M. Hallerman (Eds.), *Genetic Resources of Neotropical Fishes* (pp. 55–117). Springer International Publishing, https://doi.org/10.1007/978-3-319-55838-7

Cham, Switzerland.

Jiménez-Segura, L. F., Galvis-Vergara, G., Cala-Cala, P., García-Alzate, C. A., López-Casas, S., Ríos-Pulgarín, M. I., Arango, G. A., Mancera-Rodríguez, N. J., Gutiérrez-Bonilla, F., & Álvarez-León, R. (2016). Freshwater fish faunas, habitats and conservation challenges in the Caribbean river basins of north-western South America. *Journal of Fish Biology*, *89*(1), 65–101. https://doi.org/10.1111/jfb.13018

Jiménez-Segura, L. F., Restrepo-Santamaría, D., López-Casas, S., Delgado, J., Valderrama, M., Álvarez, J., & Gómez, D. (2014). Ictiofauna y desarrollo del sector hidroeléctrico en la cuenca del río Magdalena-Cauca, Colombia. *Biota Colombiana*, *15*(2), 3–25. https://doi.org/10.21068/bc.v15i2.317

Jombart, T. (2008). Adegenet: A R package for the multivariate analysis of genetic markers. *Bioinformatics*, *24*(11), 1403–1405. https://doi.org/10.1093/bioinformatics/btn129

Jorde, P. E., & Ryman, N. (2007). Unbiased estimator for genetic drift and effective population size. *Genetics*, *177*(2), 927–935. https://doi.org/10.1534/genetics.107.075481

Jost, L., Archer, F., Flanagan, S., Gaggiotti, O., Hoban, S., & Latch, E. (2018). Differentiation measures for conservation genetics. *Evolutionary Applications*, *11*(7), 1139–1148. https://doi.org/10.1111/eva.12590

Laikre, L., Schwartz, M. K., Waples, R. S., & Ryman, N. (2010). Compromising genetic diversity in the wild: Unmonitored large-scale release of plants and animals. *Trends in Ecology and Evolution*, *25*(9), 520–529. https://doi.org/10.1016/j.tree.2010.06.013

Landguth, E. L., Cushman, S. A., Schwartz, M. K., Mckelvey, K. S., Murphy, M., & Luikart, G. (2010). Quantifying the lag time to detect barriers in landscape genetics. *Molecular Ecology*, *19*(19), 4179–4191. https://doi.org/10.1111/j.1365-294X.2010.04808.x

Landínez-García, R. M., & Márquez, E. J. (2016). Development and characterization of 24 polymorphic microsatellite loci for the freshwater fish *Ichthyoelephas longirostris* (Characiformes: Prochilodontidae). *PeerJ*, *4*(9), e2419. https://doi.org/10.7717/peerj.2419

Li, Y. L., & Liu, J. X. (2018). StructureSelector: A web-based software to select and visualize the optimal number of clusters using multiple methods. *Molecular Ecology Resources*, *18*(1), 176–177. https://doi.org/10.1111/1755-0998.12719

Liermann, C. R., Nilsson, C., Robertson, J., & Ng, R. Y. (2012). Implications of dam obstruction for global freshwater fish diversity. *BioScience*, *62*(6), 539–548. https://doi.org/10.1525/bio.2012.62.6.5

López-Casas, S., Jiménez-Segura, L. F., Agostinho, A. A., & Pérez, C. M. (2016). Potamodromous migrations in the Magdalena River basin: bimodal reproductive patterns in neotropical rivers. *Journal of Fish Biology*, *89*(1), 157–171. https://doi.org/10.1111/jfb.12941

López-Casas, S., Rondón-Martínez, Y. F., Gutiérrez-Cortés, A., Escobar-Cardona, J. L., Muñoz-Duque, S., Valencia-Rodríguez, D., Petry, P., Batista-Morales, A. M., Rincón, C., Casas, L. F., Ospina-Pabón, J. G., Atencio-García, V., Valderrama Barco, M., Lasso, C. A., & Jiménez-Segura, L. F. (2020). Diagnóstico del grado de amenaza y medidas de manejo para los peces del río Magdalena, Colombia. In L. Jiménez-Segura & C. A. Lasso (Eds.), *XIX. Peces de la cuenca del río Magdalena, Colombia: diversidad, conservación y uso sostenible* (pp. 391–429). Serie Editorial Recursos Hidrobiológicos y Pesqueros Continentales de Colombia. Instituto de Investigación de Recursos Biológicos Alexander von Humboldt. https://doi.org/10.21068/B2020RRHHXIX10

Márquez, E., Restrepo-Escobar, N., Yepes-Acevedo, A. J., & Narváez, J. C. (2020). Diversidad y estructura genética de los peces de la cuenca del Magdalena, Colombia. In L. Jiménez-Segura & C. A. Lasso (Eds.), *XIX. Peces de la cuenca del río Magdalena, Colombia: diversidad, conservación y uso sostenible* (pp. 115–157). Serie Editorial Recursos Hidrobiológicos y Pesqueros Continentales de Colombia. Instituto de Investigación de Recursos Biológicos Alexander von Humboldt. https://doi.org/10.21068/B2020RRHHXIX03

Martinez, A. S., Willoughby, J. R., & Christie, M. R. (2018). Genetic diversity in fishes is influenced by habitat type and life-history variation. *Ecology and Evolution*, *8*(23), 12022–12031. https://doi.org/10.1002/ECE3.4661

Meirmans, P. G. (2006). Using the AMOVA framework to estimate a standardized genetic differentiation measure. *Evolution*, *60*(11), 2399–2402. https://doi.org/10.1111/j.0014-3820.2006.tb01874.x

Meirmans, P. G., & Hedrick, P. W. (2011). Assessing population structure: FST and related measures. *Molecular Ecology Resources*, *11*(1), 5–18. https://doi.org/10.1111/j.1755-0998.2010.02927.x

Mojica, J. I., Usma, J. S., Álvarez-León, R., & Lasso, C. A. (2012). *Libro rojo de peces dulceacuícolas de Colombia 2012*. Instituto de Investigación de Recursos Biológicos Alexander von Humboldt, Instituto de Ciencias Naturales de la Universidad Nacional de Colombia, WWF Colombia y Universidad de Manizales.

Mojica, J., Valderrama, M., Jimenez-Segura, L., & Alonso, J. C. (2016). *Pseudoplatystoma magdaleniatum. The IUCN Red List of Threatened Species 2016: e.T58439165A61474168.* https://dx.doi.org/10.2305/IUCN.UK.2016-1.RLTS.T58439165A61474168.en

Paetkau, D., Slade, R., Burden, M., & Estoup, A. (2004). Genetic assignment methods for the direct, real-time estimation of migration rate: A simulation-based exploration of accuracy and power. *Molecular Ecology*, *13*(1), 55–65. https://doi.org/10.1046/j.1365-294X.2004.02008.x

Peakall, R., & Smouse, P. E. (2006). GENALEX 6: Genetic analysis in Excel. Population genetic software for teaching and research. *Molecular Ecology Notes*, *6*(1), 288–295. https://doi.org/10.1111/j.1471-8286.2005.01155.x

Peakall, R., & Smouse, P. E. (2012). GenALEx 6.5: Genetic analysis in Excel. Population genetic software for teaching and research-an update. *Bioinformatics*, *28*(9), 2537–2539. https://doi.org/10.1093/bioinformatics/bts460

Pereira, L. H. G., Foresti, F., & Oliveira, C. (2009). Genetic structure of the migratory catfish *Pseudoplatystoma corruscans* (Siluriformes: Pimelodidae) suggests homing behaviour. *Ecology of Freshwater Fish*, *18*(2), 215–225. https://doi.org/10.1111/j.1600-0633.2008.00338.x

Piry, S., Alapetite, A., Cornuet, J.-M., Paetkau, D., Baudouin, L., & Estoup, A. (2004). GENECLASS2: A software for genetic assignment and first-generation migrant detection. *Journal of Heredity*, *95*(6), 536–539. https://doi.org/10.1093/jhered/esh074

Piry, S., Luikart, G., & Cornuet, J. M. (1999). BOTTLENECK: A computer program for detecting recent reductions in the effective population size using allele frequency data. *Journal of Heredity*, *90*(4), 502–503. https://doi.org/10.1093/jhered/90.4.502

Prado, F. D., Fernandez-Cebrián, R., Foresti, F., Oliveira, C., Martínez, P., & Porto-Foresti, F. (2018). Genetic structure and evidence of anthropogenic effects on wild populations of two Neotropical catfishes: baselines for conservation. *Journal of Fish Biology*, *92*(1), 55–72. https://doi.org/10.1111/jfb.13486

Pritchard, J. K., Stephens, M., & Donnelly, P. (2000). Inference of population structure using multilocus genotype data. *Genetics*, *155*(2), 945–959.

Puechmaille, S. J. (2016). The program STRUCTURE does not reliably recover the correct population structure when sampling is uneven: subsampling and new estimators alleviate the problem. *Molecular Ecology Resources*, *16*(3), 608–627. https://doi.org/10.1111/1755-0998.12512

Raymond, M., & Rousset, F. (1995). GENEPOP (Version 1.2): Population genetics software for exact tests and ecumenicism. *Journal of Heredity*, *86*(3), 248–249. https://doi.org/10.1093/oxfordjournals.jhered.a111573

Reid, A. J., Carlson, A. K., Creed, I. F., Eliason, E. J., Gell, P. A., Johnson, P. T. J., Kidd, K. A., MacCormack, T. J., Olden, J. D., Ormerod, S. J., Smol, J. P., Taylor, W. W., Tockner, K., Vermaire, J. C., Dudgeon, D., & Cooke, S. J. (2019). Emerging threats and persistent conservation challenges for freshwater biodiversity. *Biological Reviews*, *94*(3), 849–873. https://doi.org/10.1111/brv.12480

Restrepo, J. D., Cárdenas-Rozo, A., Paniagua-Arroyave, J. F., & Jiménez-Segura, L. (2020). Aspectos físicos de la cuenca del río Magdalena, Colombia: geología, hidrología, sedimentos, conectividad, ecosistemas acuáticos e implicaciones para la biota. In L. Jiménez-Segura & C. A. Lasso (Eds.), *XIX. Peces de la cuenca del río Magdalena, Colombia: diversidad, conservación y uso sostenible* (pp. 41–83). Serie Editorial Recursos Hidrobiológicos y Pesqueros Continentales de Colombia. Instituto de Investigación de Recursos Biológicos Alexander von Humboldt. https://doi.org/10.21068/B2020RRHHXIX01

Rodríguez-Olarte, D., Mojica, J. I., & Taphorn, D. (2011). Northern South America Magdalena and Maracaibo Basins. In J. S. Albert & R. E. Reis (Eds.), *Historical Biogeography of Neotropical Freshwater Fishes.* (pp. 243–257). University of California Press.

Rousset, F. (2008). GENEPOP'007: A complete re-implementation of the GENEPOP software for Windows and Linux. *Molecular Ecology Resources*, *8*(1), 103–106. https://doi.org/10.1111/j.1471-8286.2007.01931.x

Schwartz, M. K., Luikart, G., & Waples, R. S. (2007). Genetic monitoring as a promising tool for conservation and management. *Trends in Ecology and Evolution*, *22*(1), 25–33. https://doi.org/10.1016/j.tree.2006.08.009

Valderrama, M., Escobar C., J. L., Pardo B., R., Toro S., M., Gutiérrez C., J. C., & López C., S. (2020). Servicios ecosistémicos generados por los peces de la cuenca del rio Magdalena, Colombia. In L. Jiménez-Segura & C. A. Lasso (Eds.), *XIX. Peces de la cuenca del río Magdalena, Colombia: diversidad, conservación y uso sostenible* (pp. 205–235). Serie Editorial Recursos Hidrobiológicos y Pesqueros Continentales de Colombia. Instituto de Investigación de Recursos Biológicos Alexander von Humboldt. https://doi.org/10.21068/B2020RRHHXIX05

Van Oosterhout, C., Hutchinson, W. F., Wills, D. P. M., & Shipley, P. (2004). MICRO-CHECKER: Software for identifying and correcting genotyping errors in microsatellite data. *Molecular Ecology Notes*, *4*(3), 535–538. https://doi.org/10.1111/j.1471-8286.2004.00684.x

Vega- Retter, C., Muñoz- Rojas, P., Rojas- Hernández, N., Copaja, S., Flores- Prado, L., & Véliz, D. (2020). Dammed river: Short- and long- term consequences for fish species inhabiting a river in a Mediterranean climate in central Chile. *Aquatic Conservation: Marine and Freshwater Ecosystems*, *30*(12), 2254–2268. https://doi.org/10.1002/aqc.3425

Waples, R. S. (2015). Testing for Hardy-Weinberg proportions: Have we lost the plot? *Journal of Heredity*, *106*(1), 1–19. https://doi.org/10.1093/jhered/esu062

Waples, R. S., Antao, T., & Luikart, G. (2014). Effects of overlapping generations on linkage disequilibrium estimates of effective population size. *Genetics*, *197*(2), 769–780. https://doi.org/10.1534/genetics.114.164822

Waples, R. S., & Do, C. (2010). Linkage disequilibrium estimates of contemporary Ne using highly variable genetic markers: A largely untapped resource for applied conservation and evolution. *Evolutionary Applications*, *3*(3), 244–262. https://doi.org/10.1111/j.1752-4571.2009.00104.x

Waples, R. S., & England, P. R. (2011). Estimating contemporary effective population size on the basis of linkage disequilibrium in the face of migration. *Genetics*, *189*(2), 633–644. https://doi.org/10.1534/genetics.111.132233

Yamamoto, S., Morita, K., Koizumi, I., & Maekawa, K. (2004). Genetic differentiation of white-spotted charr (*Salvelinus leucomaenis*) populations after habitat fragmentation: Spatial-temporal changes in gene frequencies. *Conservation Genetics*, *5*(4), 529–538. https://doi.org/10.1023/B:COGE.0000041029.38961.a0
